# Roe deer used as indicator species for a country wide survey for the occurrence of Tick Borne Encephalitis in Austria

**DOI:** 10.1186/1756-3305-7-S1-O17

**Published:** 2014-04-01

**Authors:** GG Duscher, R Baumgartner, G Walder

**Affiliations:** 1 Department of Pathobiology, Institute of Parasitology, Vienna, Austria; 2 Dr. Gernot Walder GmbH, Außervillgraten, Austria

## 

Although 85% of the Austrian citizens are vaccinated against Tick Borne Encephalitis (TBE), still ~100 clinical cases are reported each year. Until now, risk maps are created by using human cases only, albeit this source is biased due to the high level of vaccinated persons, uncertainties about the location of infection and undocumented cases because of inapparent infections.

To overcome this problem, several solutions are proposed: Investigations of ticks by PCR seems to be reasonable, when looking on small areas. Another possibility to gain knowledge about TBE occurrence is to investigate the sera of wildlife animals for antibodies against TBE.

Due to the fact that roe deer can be found all around the whole Austrian landscape, but its habitat is restricted to a relatively small area, we chose this species as indicators for the monitoring of antibodies against TBE.

Together with the hunting organisations blood samples from whole Austria was collected starting in September 2013 and screened for the occurrence of antibodies against TBE using an indirect immunofluorescence assay test.

A total of 928 serum samples from roe deer were collected so far. The analysis of ~100 samples showed positivity to TBE in 2 roe deer. The shooting/finding location of each roe deer was georeferenced and a geographical information system was constructed. The findings of this survey on roe deer will be used to update the TBE risk map in Austria.

